# Exploring the association between type 2 diabetes and fecal incontinence in american adults: insights from a large cross-sectional study

**DOI:** 10.1007/s00384-024-04697-6

**Published:** 2024-07-31

**Authors:** Lun-chao Li, Li-Ming Liang, Hong-ye Ji, Can Zhang, Man Wang, Hong-sheng Liu

**Affiliations:** 1 Department of Emergency Medicine, the Fourth Medical Center of PLA General Hospital, 51 Fucheng Road, Haidian District, Beijing, 100048 P.R. China; 2 Plastic and Reconstructive Surgery Unit, Department of Burns and Plastic Surgery, the Fourth Medical Center of PLA General Hospital, Beijing, 100048 P.R. China

**Keywords:** Type 2 diabetes, Fecal incontinence, National Health and Nutrition Examination, Sedentary

## Abstract

**Background:**

The relationship between fecal incontinence (FI) and type 2 diabetes (T2D) has been well recognized, but a comprehensive understanding of this relationship is lacking, taking into account demographic factors and lifestyle variables.

**Methods:**

Using a cross-sectional approach, 13,510 adults aged 20 years and older were identified from the 2005–2010 National Health and Nutrition Examination Survey. Multivariate logistic regression models were used to calculate the adjusted odds ratios (ORs), and further subgroup analyses and propensity score analysis were performed to ensure stable results.

**Results:**

Among 13,510 adults, 11.2% had T2D, and 8.8% had FI. We found a strong T2D-FI link (OR: 1.30; 95% CI: 1.09–1.54, *P* < 0.001), even after adjusting for covariates. Age > 45 was a critical factor, with a stronger T2D-FI association. Sedentary behavior (OR: 1.41; 95% CI: 1.15–1.73) in T2D patients were associated with FI.

**Conclusions:**

Our study highlights the significant T2D-FI link in US adults, especially in older T2D patients. Lifestyle changes may reduce FI risk. More research is needed for causality and mechanisms.

**Supplementary Information:**

The online version contains supplementary material available at 10.1007/s00384-024-04697-6.

## Background

Fecal incontinence (FI) is characterized by the unexpected loss of liquid, solid, or mucous feces occurring at least once a month, leading to a subjective inability to control bowel movements [1, 2]. Studies since 1995 have highlighted the prevalence of FI in both nursing homes and the community, with a global prevalence of ≤ 7% among community-dwelling adults, significantly impacting their quality of life [3]. In the United States, FI affects 8.39% of non-hospitalized adults, with an estimated annual direct cost of $17,166 per person [4]. Despite its substantial burden, there remains limited understanding of the risk factors for FI and the potential for targeted prevention strategies [3]. Some studies have identified individuals with diabetes as a high-risk group for FI [5]. Currently, one in ten adults has diabetes mellitus, with predictions suggesting that one in three adults will have type 2 diabetes by 2050 [6–8].

Previous research has demonstrated that FI is significantly associated with high bowel frequency among women with diabetes [9]. Another prospective study in older women revealed that higher levels of physical activity were linked to a 25% reduction in FI risk [10]. However, there is a lack of studies examining the relationship between type 2 diabetes (T2D) and FI in nationally representative samples. Furthermore, it remains unclear whether this association varies among subgroups based on factors such as age, sex, lifestyle, and common comorbidities. Therefore, this study utilized data from the National Health and Nutrition Examination Survey (NHANES) to investigate the association between T2D and FI, considering factors such as age, comorbidities, body weight, and physical activity consistency, in a representative sample of non-hospitalized adults aged 20 to 85 years. The identification of high-risk subgroups within the T2D population is crucial for targeted preventive interventions.

This study used data from the nation's largest National Health and Nutrition Examination Survey conducted in 2005––2010 because the digestive health questionnaire only included information for these three two-year time periods, all of which came from the official website 1, which is based on NHANES data available to the general public, which included detailed data on demographics, health behaviors, and medical comorbidities, primarily to determine whether T2D was associated with FI reporting. The association between T2D and other known FI-related factors, such as age, comorbidities, body weight, and physical activity consistency, was assessed in a representative sample of non-hospitalized adults aged 20–85 years.

## Methods

### Data sources and study population

This study utilized data from the National Health and Nutrition Examination Survey (NHANES), a comprehensive survey designed to assess the health and nutritional status of non-institutionalized individuals in the United States. The NHANES employs a multistage stratified probability sampling method to select participants, ensuring representation of the U.S. population across different demographic groups [11, 12].

Data for this analysis were drawn from NHANES surveys conducted between 2005 and 2010, as these cycles included detailed information on demographics, health behaviors, and medical comorbidities. Participants in this study were required to be aged 20 years or older and had completed interviews and evaluations at mobile examination centers (MECs). All participants provided informed consent, and the study was approved by the NCHS Ethics Review Committee. Subjects with missing data on FI, covariates, or T2D status were excluded from the analysis.

### FI assessment

Fecal incontinence (FI) was assessed using the Bowel Health Questionnaire, which collected information on any unintentional bowel leakage that occurred within the past month, including episodes of solid stool, gas, liquid, or mucus leaks [13, 14]. Participants reported the frequency of these events, ranging from "never" to "once a day or more." FI was defined as any involuntary mucous, liquid, or solid stool loss occurring within the past 30 days, categorized as either "Yes" or "No" for analysis [15–17].

### T2D assessment

The presence of type 2 diabetes (T2D) was determined based on self-report questionnaires administered before the physical examination [18]. Participants were classified as having T2D if they reported a prior diagnosis of T2D by a physician, in accordance with the American Diabetes Association criteria [19].

### Other covariates

A range of covariates were considered in this study, including age, sex, marital status, race/ethnicity, body mass index (BMI), education level, poverty income ratio (PIR), smoking status, drinking status, and physical activity. BMI was calculated as weight in kilograms divided by height in meters squared, categorized into three groups using thresholds of 25 and 30 kg/m^2^. Smoking status was categorized as current smokers, former smokers, and never smokers. Participants who had never smoked or had smoked fewer than 100 cigarettes were categorized as "Unknown never smokers." Drinking status was defined based on the reported consumption of 12 or more alcoholic drinks annually, classifying participants as drinkers or non-drinkers.

Additionally, common comorbidities such as liver disease, heart disease, lung disease, hypertension, arthritis, and cancer were assessed using specific questions and dichotomous variables.

### Statistical analyses

Descriptive analyses were conducted to summarize participant characteristics. Continuous data were presented as means with standard deviations, while categorical variables were expressed as proportions (%). Chi-square tests and t-tests were used to compare categorical and continuous variables, respectively.

Logistic regression models were employed to examine the association between T2D and FI.The models included both non-adjusted and multivariate-adjusted models, progressively accounting for demographic factors, health behaviors, and comorbidities. Subgroup analyses were performed to assess the stability of the T2D-FI association across different demographic and clinical subgroups.All statistical analyses were performed using R (version http://www.R-project.org) and Free Statistics software (version 1.8), with statistical significance defined as *p* < 0.05.

We conducted a series of sensitivity analyses to evaluatethe robustness of the findings of the study and how ourconclusions can be affected by applying various asso-ciation inference models. In the sensitivity analysis, weapplied four more association inference models: a doubly robust model adjusting for all covariates, a propen-sity score-based IPW model, a propensity score-basedpatient-matching model, and a logistic regression-basedmultivariate analysis model. The calculated effect sizesand p values from all these models were reported andcompared.This study adhered to STROBE Guidelines of reporting.

## Results

### Study population

**Fig. 1:**
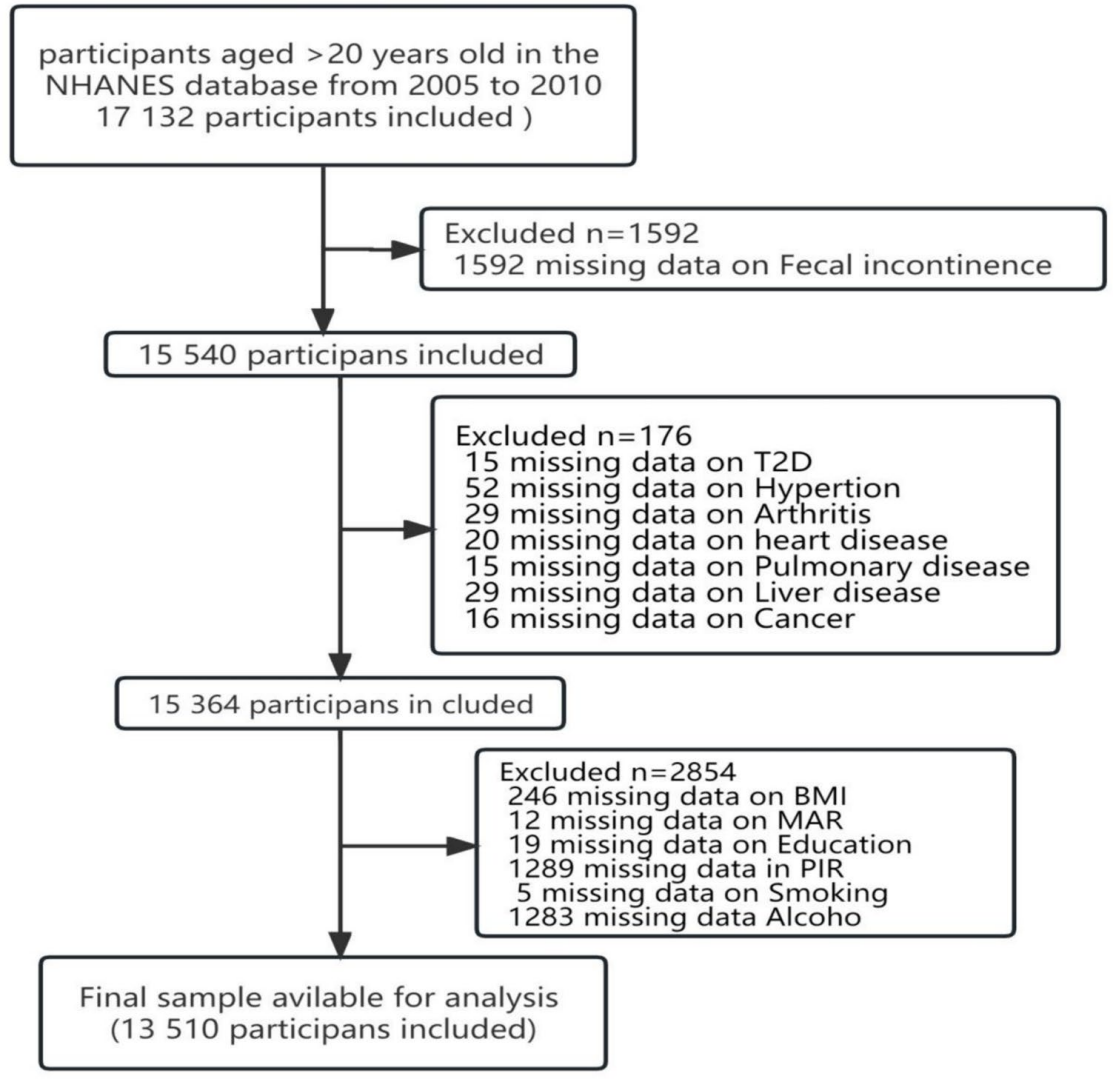
Illustrates the exclusion process. A total of 17,132 adults aged 20 years or older who completed interviews and underwent screening at mobile examination centers (MECs) were initially included in this study. After excluding participants, a final sample of 13,510 participants was included in the analysis

A total of 17,132 adults aged 20 years or older who completed interviews and underwent screening at mobile examination centers (MECs) were initially included in this study. After excluding participants with deficient fecal incontinence (FI) (*n* = 592) and those with missing covariate data (*n* = 3,030), a final sample of 13,510 participants was included in the analysis. Figure 1 illustrates the exclusion process.

### Baseline characteristics

Table 1 presents the baseline characteristics of the study participants categorized by the presence or absence of type 2 diabetes (T2D). Among the participants, 1,518 individuals (11.2%) reported having T2D. The average age of the study participants was 49.1 years (standard deviation: 18.0), with 6,883 (50.9%) being female. A total of 1,192 participants (8.8%) reported experiencing FI, with 237 (15.6%) of them having T2D, which was significantly higher than the 955 (8.0%) individuals without T2D (*P* < 0.001). Participants aged over 45 years, of white ethnicity, widowed or separated, physically inactive, and obese had higher rates of T2D and were more likely to have other comorbidities, including high blood pressure, arthritis, heart disease, lung disease, liver disease, and cancer.

**Table 1:** Presents the baseline characteristics of the study participants categorized by the presence or absence of type 2 diabetes (T2D)

Variables	Total	Without T2D	With T2D	*P*
	*n* = 13,510	*n* = 11,992	*n* = 1518	
Age(year)	49.1 ± 18.0	47.6 ± 17.9	61.5 ± 12.9	< 0.001
Gender				0.203
male	6627 (49.1)	5859 (48.9)	768 (50.6)	
female	6883 (50.9)	6133 (51.1)	750 (49.4)	
Race				< 0.001
Mexican American	2399 (17.8)	2100 (17.5)	299 (19.7)	
Other Hispanic	1053 ( 7.8)	925 (7.7)	128 (8.4)	
Non-Hispanic White	6799 (50.3)	6192 (51.6)	607 (40.0)	
Non-Hispanic Black	2699 (20.0)	2270 (18.9)	429 (28.3)	
Other Race-Including Multi-Racial	560 ( 4.1)	505 (4.2)	55 (3.6)	
Marital				< 0.001
Married	7255 (53.7)	6398 (53.4)	857 (56.5)	
Widowed	1117 ( 8.3)	881 (7.3)	236 (15.5)	
Divorced	1469 (10.9)	1250 (10.4)	219 (14.4)	
Separated	434 ( 3.2)	376 (3.1)	58 (3.8)	
Never married	2180 (16.1)	2072 (17.3)	108 (7.1)	
Living with partner	1055 ( 7.8)	1015 (8.5)	40 (2.6)	
Education				< 0.001
≤ high school	6950 (51.4)	5987 (49.9)	963 (63.4)	
> high school	6560 (48.6)	6005 (50.1)	555 (36.6)	
Family PIR	2.6 ± 1.6	2.6 ± 1.6	2.3 ± 1.5	< 0.001
Smoking				< 0.001
never	7088 (52.5)	6348 (52.9)	740 (48.7)	
former	3444 (25.5)	2911 (24.3)	533 (35.1)	
Current	2978 (22.0)	2733 (22.8)	245 (16.1)	
Alcohol				< 0.001
Yes	9634 (71.3)	8722 (72.7)	912 (60.1)	
Hypertension				< 0.001
Yes	3865 (28.6)	2943 (24.5)	922 (60.7)	
Arthritis				< 0.001
Yes	3703 (27.4)	2957 (24.7)	746 (49.1)	
Heart.disease				< 0.001
Yes	1152 ( 8.5)	780 (6.5)	372 (24.5)	
Pulmory.disease				< 0.001
Yes	2385 (17.7)	2023 (16.9)	362 (23.8)	
Liver.disease				< 0.001
Yes	452 ( 3.3)	363 (3.0)	89 (5.9)	
Cancer				< 0.001
Yes	1292 ( 9.6)	1078 (9.0)	214 (14.1)	
BMI(kg/m^2^)	29.1 ± 6.8	28.6 ± 6.6	32.6 ± 7.6	< 0.001
activity				< 0.001
Inactive	6689 (49.5)	5687 (47.4)	1002 (66.0)	
Moderate	3716 (27.5)	3322 (27.7)	394 (26.0)	
Vigorous	3105 (23.0)	2983 (24.9)	122 (8.0)	
FI,n (%)				< 0.001
Yes	1192 ( 8.8)	955 (8.0)	237 (15.6)	

### Association between T2D and FI

Univariate logistic regression analysis (Table 2) revealed that age, sex, race/ethnicity (non-Hispanic white and non-Hispanic black), living arrangements, overweight status, smoking, comorbidities, diabetes, and other variables were significantly associated with a higher probability of FI (*P* < 0.05). In contrast, participants who were married, cohabiting, and engaged in high, moderate, or vigorous physical activity had a reduced probability of FI (*P* < 0.05).

Table 2 displays the results of logistic multivariate regression models examining the association between T2D and FI. In the unadjusted model (Model 1), T2D was strongly associated with an increased risk of FI (odds ratio [OR]: 2.14, 95% confidence interval [CI]: 1.83–2.49, *P* < 0.001). This association remained significant after adjusting for demographics (Model II), risk behaviors (Model III), and common comorbidities (Model IV), with ORs of 1.54 (95% CI: 1.31–1.80, *P* < 0.001), 1.58 (95% CI: 1.34–1.85, *P* < 0.001), and 1.36 (95% CI: 1.15–1.61, *P* < 0.001), respectively. Even after further adjustment for BMI and physical activity (Model V), the association between T2D and FI remained significant (OR: 1.30, 95% CI: 1.09–1.54, *P* < 0.001).

**Table 2:** Multivariate regression analysis of the association between Type 2 diabetes and fecal incontinence

Variable	OR_95CI	adj.P_value
Model I	2.14 (1.83–2.49)	< 0.001
Model II	1.54 (1.31–1.80)	< 0.001
Model III	1.58(1.34–1.85)	< 0.001
Model IV	1.36 (1.15–1.61)	< 0.001
Model V	1.30 (1.09–1.54)	< 0.001

Model I: no adjusted

Model II: adjusted for age + sex + race/ethnicity + marital status + education level + PIR

Model III: Model II + smoking status + alcohol consumption

Model IV: Model III + hypertension + arthritis + heart disease + pulmonary disease + liver disease + cancer

Model V: Model IV + BMI + activity

### Sensitivity analyses of association between T2D and FI

For the sensitivity analysis, as summarized in Table 3, all six estimation models (propensity score adjusted [PSA], propensity score matching [PSM], the inverse probability of treatment weighting [IPTW], the standardized mortality ratio weighting [SMRW], pairwise algorithmic [PA], overlap weight [OW]) led to the same conclusion: patients who had T2D had higher fecal incontinence risk (OR range 1.28 to 1.43, all P value < 0.005).

**Table 3:** Sensitivity Analyses of association of T2Dand fecal incontinence risk

Item1	Diabetes	OR_95CI	P_value
PropensityScore adjusted	Yes vs NO	1.43 (1.2–1.69)	< 0.001
PropensityScore Matched	Yes vs NO	1.42 (1.15–1.75)	0.001
Weighted IPTW	Yes vs NO	1.28 (1.06–1.53)	0.009
Weighted SMRW	Yes vs NO	1.36 (1.17–1.58)	< 0.001
Weighted PA	Yes vs NO	1.34 (1.09–1.66)	0.006
Weighted Ow	Yes vs NO	1.35 (1.06–1.72)	0.017

**Fig. 2:**
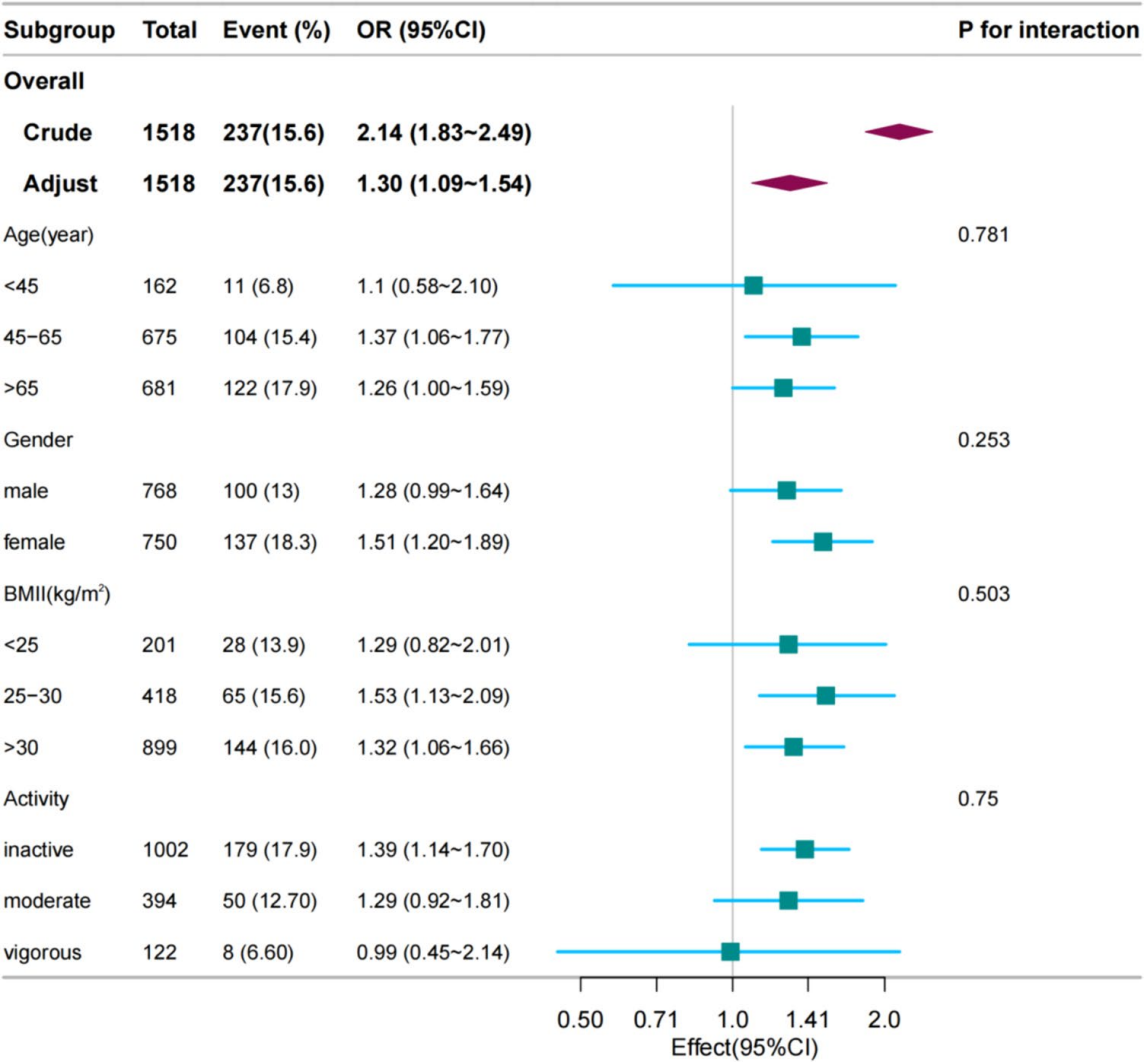
Association between Type 2 diabetes and fecal incontinence. Subgroup analysis (Fig. 2) did not reveal statistically significant interactions based on age, BMI, physical activity, or smoking status (all P > 0.05). Notes: Adjusted for age, sex, race/ethnicity, marital status, education level, Family PIR, smoking status, alcohol consumption,, liver disease,heart disease, pulmonary disease, hypertension, arthritis and cancer

The subgroup analysis of the data is shown in Fig. 2. No statistically significant interactions were observed in the subgroup analyses according to age, Gender,BMI and physical activity (all P > 0.05). In two subgroups of subjects aged 45 years and older, T2D was significantly associated with FI, with an odds ratio (OR) and a 95% CI of 1.37(1.06–1.77) and 1.26(1.0–1.59), respectively. In the Gender,BMI and physical activity subgroups, no association was shown between T2D participants and FI in men, BMI < 25, and moderate and vigorous exercise subgroups.

## Discussion

This comprehensive study unveils several crucial facets of the relationship between type 2 diabetes (T2D) and fecal incontinence (FI) within a large and nationally representative cohort of American adults. Our findings carry significant implications for both clinical practice and the direction of future research.

Our study reaffirms and strengthens the well-established link between T2D and FI. Notably, this association remains substantial even after meticulous adjustments for various demographic variables, lifestyle factors, and with common comorbidities [20–22]. Specifically, our results indicate that T2D participants are associated an increased risk of FI (OR, 1.30; 95% CI: 1.09–1.54, *P* < 0.001). This robust connection underscores the clinical relevance of T2D as a meaningful risk factor for FI, suggesting that healthcare providers should integrate FI assessments as part of routine care for individuals with T2D.

One of our noteworthy observations is the significant interaction between age and the T2D-FI relationship. Individuals aged 45 years and older exhibit a particularly strong association between T2D and FI, with an odds ratio (OR) and a 95% CI of 1.37(1.06–1.77) and 1.26(1.0–1.59), respectively.This finding underscores age as a critical modifier in this relationship. The increased vulnerability to FI among older adults with T2D emphasizes the need for targeted screening and intervention efforts within this demographic. Age-related changes in pelvic floor muscles and other factors may contribute to the heightened susceptibility to FI in this age group [22–25].

Our study provides valuable insights into the role of lifestyle factors in shaping the T2D-FI connection. Sedentary behavior emerges as a potent risk factor for FI in individuals with T2D, with an OR and 95% CI of 1.41(1.15–1.73, P = 0.001). Encouraging T2D patients to adopt a physically active lifestyle may prove instrumental in mitigating the risk of FI.

Although previous studies have yielded varied results regarding the relationship between Body Mass Index (BMI) and Fecal Incontinence (FI), our study introduces new insights. We found that in the population with Type 2 Diabetes (T2D), participants with a BMI lower than 25 were not associated with FI (OR: 1.29, 95% CI: 0.82–2.01, *P* < 0.001). This lack of association in the T2D population with FI might be due to the lower incidence of diabetes among individuals with low body weight. While the mechanisms behind obesity predisposing individuals to FI are not entirely clear, obesity is a risk factor for diarrhea and accelerated colonic transit, possibly related to increased intra-abdominal pressure (which can damage the pelvic floor) and rectal pressures [23, 26, 27]. It is noteworthy that our study results demonstrate a strong association between FI and overweight and obese individuals within the T2D population [10, 17, 28] and suggests that patients with T2D and FI might benefit from modifying lifestyle factors such as exercise and reducing BMI, and further research is needed to understand the benefits of life style in improving patients with T2D and FI.In this study, we reported 8.8% participants suffering from fecal incontinence in T2D, which may be underreported in general population. FI should be screened in people with other chronic disease, not only in T2D.

### Strengths and limitations

This study's strengths lie in its extensive, nationally representative dataset and the meticulous consideration of various covariates. By examining the T2D-FI relationship across diverse demographic and clinical subgroups, we provide a nuanced understanding of this connection. Nevertheless, our study is not without limitations, inherent to cross-sectional analyses, such as the inability to establish causation and susceptibility to reverse causality. Furthermore, the reliance on data from over a decade ago and self-reported medical conditions introduces potential reporting bias.

### Clinical implications

The findings from this study carry essential implications for clinical practice. Healthcare providers should recognize the heightened risk of FI in individuals with T2D and incorporate FI assessments into routine evaluations, particularly for those aged 45 years and older. Encouraging lifestyle modifications, including increased physical activity and losing weight, can be pivotal for reducing FI risk in T2D patients, ultimately enhancing their quality of life.

### Future research directions

Prospective research endeavors are warranted to elucidate causality and delve into the underlying mechanisms governing the T2D-FI relationship. Furthermore, investigations into the effectiveness of lifestyle interventions, such as structured exercise programs, in mitigating FI risk among T2D patients are of paramount importance. Longitudinal studies that account for dynamic changes in T2D management and comorbidities over time can provide a more nuanced understanding of FI risk factors in this population.

## Conclusion

Our study reinforces the substantial connection between T2D and FI while shedding light on the role of age, lifestyle factors, and BMI in shaping this relationship. These findings underscore the imperative of targeted screening and intervention strategies for FI in T2D patients, particularly among older adults and those with sedentary lifestyles. Lifestyle modifications emerge as promising avenues for reducing FI risk among individuals grappling with both T2D and its associated complications, as highlighted by the data-driven correlations we've presented.

## Supplementary Information

Below is the link to the electronic supplementary material.

Supplementary file1 (DOCX 33 KB)

Table 2 Univariate regression analysis of the association between Type 2 diabetes and fecal incontinence. Univariate logistic regression analysis (Table 2) revealed that age, sex, race/ethnicity (non-Hispanic white and non-Hispanic black), living arrangements, overweight status, smoking, comorbidities, diabetes, and other variables were significantly associated with a higher probability of FI (*P* < 0.05) (DOCX 14 KB)

## Data Availability

No datasets were generated or analysed during the current study.

## Declarations

### Approval of the research protocol

The CDC Institutional Research Ethics Review Committee approved the protocol for the NHANES.

### Animal studies

N/A
