# Supplementary material for: Exploring the association between type 2 diabetes and fecal incontinence in american adults: insights from a large cross-sectional study
Source: Int J Colorectal Dis. 2024 Jul 31;39(1):121. doi: 10.1007/s00384-024-04697-6 (PMC11291540; doi:10.1007/s00384-024-04697-6)
Supplement: Supplementary file 2 — Table 2 Univariate regression analysis of the association between Type 2 diabetes and fecal incontinence. Univariate logistic regression analysis (Table 2) revealed that age, sex, race/ethnicity (non-Hispanic white and non-Hispanic black), living arrangements, overweight status, smoking, comorbidities, diabetes, and other variables were significantly associated with a higher probability of FI (P < 0.05) (DOCX 14 KB) [file 384_2024_4697_MOESM2_ESM.docx]

Table 2 .Univariate regression analysis of the association between Type 2 diabetes and fecal incontinence.

| Variable | OR_95CI | P_value |
| --- | --- | --- |
| Age(year): 45-65 vs < 45 | 2.34 (1.98-2.77) | <0.001 |
| Age(year): >65 vs < 45 | 3.32 (2.86-3.85) | <0.001 |
| Gender: Female vs Male | 1.25 (1.11-1.41) | <0.001 |
| Race/Ethnicity: ref. = Mexican American |  |  |
| Other Hispanic | 1.1 (0.83-1.47) | 0.502 |
| Non-Hispanic White | 1.64 (1.37-1.96) | <0.001 |
| Non-Hispanic Black | 1.28 (1.03-1.58) | 0.025 |
| Other Race-Including Multi-Racial | 1.29 (0.91-1.81) | 0.149 |
| Marital Status: Widowed vs Married | 1.7 (1.4-2.05) | <0.001 |
| Marital Status: Divorced vs Married | 1.47 (1.23-1.75) | <0.001 |
| Marital Status: Separated vs Married | 1.56 (1.16-2.1) | 0.002 |
| Marital Status: Never married vs Married | 0.72 (0.6-0.88) | <0.001 |
| Marital Status: Living with partner vs Married | 0.6 (0.46-0.8) | 0.001 |
| Education Level: > high school vs ≤ high school | 0.9 (0.8-1.01) | 0.071 |
| Family PIR | 0.93 (0.89-0.96) | <0.001 |
| BMI(kg/m^2^): 25-30 vs <25 | 1.09 (0.93-1.28) | 0.286 |
| BMI(kg/m^2^): >30 vs <25 | 1.40 (1.21-1.63) | <0.001 |
| activity: Moderate vs Inactive | 0.78 (0.68-0.89) | <0.001 |
| activity: Vigorous vs Inactive | 0.39 (0.32-0.47) | <0.001 |
| Smoking: former vs Never | 1.52 (1.32-1.74) | <0.001 |
| Smoking: Currentvs Never | 1.24 (1.06-1.44) | 0.006 |
| Alcohol: Yes vs No | 0.88 (0.78-1.01) | 0.06 |
| Hypertension: Yes vs No | 2.08 (1.84-2.34) | <0.001 |
| Arthritis: Yes vs No | 2.25 (1.99-2.54) | <0.001 |
| Heart.disease: Yes vs No | 2.56 (2.18-3.02) | <0.001 |
| Pulmonary.disease: Yes vs No | 1.68 (1.47-1.93) | <0.001 |
| Liver.disease: Yes vs No | 1.61 (1.22-2.13) | 0.001 |
| Cancer: Yes vs No | 2.01 (1.71-2.38) | <0.001 |
| Diabetes: Yes vs No | 2.14 (1.83-2.49) | <0.001 |
